# Supplementary material for: The Inventory of Personality Organization-Reality Testing Subscale and Belief in Science Scale: Confirmatory factor and Rasch analysis of thinking style measures
Source: PLoS One. 2024 Sep 6;19(9):e0310055. doi: 10.1371/journal.pone.0310055 (PMC11379196; doi:10.1371/journal.pone.0310055)
Supplement: Attachment — Submitted filename: Response to Reviewers.docx [file pone.0310055.s002.docx]

**Comments to the Author**

Review Comments to the Author

Please use the space provided to explain your answers to the questions above. You may also include additional comments for the author, including concerns about dual publication, research ethics, or publication ethics. (Please upload your review as an attachment if it exceeds 20,000 characters)

Reviewer #1: Dear authors,

The manuscript is good enough, it only needs a few additional figures in several parts. For example, a flowchart can be added to the introduction, as well as examples for research instruments, namely the Inventory of Personality Organization-Reality Testing Subscale (IPO-RT) and the Belief in Science Scale (BIS).

Best regards,

Response:

Thank you for your feedback. We have included a figure with a flowchart of the research phases. Please note that we originally included examples of items from the IPO-RT and BIS. In terms of your other points, we have included a recommendation in the Abstract, and stated the research objectives more explicitly. Please note that we mentioned in the original manuscript when the research was conducted.

Reviewer #2: Very interesting this article.Tthe study reveals adequate methodological capacity. The theoretical framework refers the reader to studies on the variables that are appropriate to the research objectives. The presentation of the data appears to be consistent, based on the methodological and ethical definition. The discussion and conclusions also appear to be consistent.
-Proposal to improve bibliographical references in accordance with APA standards, for example reference nº66

Response:

Thank you for your supportive review of the manuscript. Please note that we followed the PLOS One referencing style requirements for the manuscript (Vancouver).
